# Laparoscopic total biopsy for suspected gallbladder cancer: A case series

**DOI:** 10.1002/hsr2.156

**Published:** 2020-04-20

**Authors:** Yukio Tokumitsu, Yoshitaro Shindo, Hiroto Matsui, Satoshi Matsukuma, Masao Nakajima, Shin Yoshida, Michihisa Iida, Nobuaki Suzuki, Shigeru Takeda, Hiroaki Nagano

**Affiliations:** ^1^ Department of Gastroenterological, Breast and Endocrine Surgery Yamaguchi University Graduate School of Medicine Ube Yamaguchi Japan

**Keywords:** gallbladder bed dissection, gallbladder cancer, laparoscopic surgery, Total biopsy, whole‐layer cholecystectomy

## Abstract

**Background and aims:**

Imaging diagnosis of gallbladder cancer remains difficult to achieve preoperatively. We developed a novel approach based on laparoscopic whole‐layer cholecystectomy (LWLC) and laparoscopic gallbladder bed dissection (LGBD) for total biopsy, for ultimately determining the optimal treatment strategy for suspected gallbladder cancer detected on preoperative imaging. Here, we describe a case series of patients who underwent this procedure at our institution.

**Methods:**

We retrospectively examined clinicopathological data of consecutive patients with suspected gallbladder carcinoma at Yamaguchi University Graduate School of Medicine from September 2016 to July 2018 on which a laparoscopic approach was used. Preoperative imaging findings suggestive of gallbladder cancer were defined as follows: elevated lesion >10 mm in diameter, increasing tumor size over time compared with the previous imaging, sessile lesion, irregular wall thickness lesion mimicking cancer, elevated lesion with dense enhancement, or positive results on fluorodeoxyglucose positron emission tomography. LWLC was performed for early‐stage or suspected malignant lesions without liver invasion, and LGBD was performed for lesions with an unclear border between the gallbladder and the liver. When postoperative pathological examination revealed the presence of gallbladder cancer invading into the subserosal layer, additional gallbladder bed resection and regional lymphadenectomy were considered. Patient characteristics, perioperative findings, pathological findings, and postoperative outcomes of patients who underwent LWLC or LGBD were reviewed retrospectively, and the short‐term outcomes of the laparoscopic approach were analyzed.

**Results:**

Fifteen consecutive patients were included in the study. The median age of the patients was 63 years (IQR 42‐76 years); 7 patients were males. We performed LWLC in 12 cases and LBGD in 3 cases. Median (IQR) operation time was 159 (140‐193) min and median blood loss was 10 (5–30) mL. No bile leakage caused by intraoperative perforation of the gallbladder was seen. Median hospital stay was 7 (5–9) days. Only one patient developed postoperative complications (abdominal abscess). Histologically, gallbladder cancer was diagnosed in five cases (pT1a, n = 2; pT2, n = 3), and two of the pT2 patients underwent additional open surgery.

**Conclusions:**

Our laparoscopic‐based approach for suspected gallbladder cancer might represent a safe strategy and could play an important role in defining the optimal treatment strategy.

## 1 INTRODUCTION

Although laparoscopic cholecystectomy is a standard approach for benign lesions such as cholecystolithiasis, laparoscopic surgery for suspected gallbladder cancer has not been widely accepted because of the potential for peritoneal dissemination and port‐site recurrence (PSR) by intraoperative gallbladder perforation of the thinned gallbladder wall.^1^, ^2^ On the other hand, definitive diagnosis of gallbladder cancer and determination of the exact depth of cancer invasion remain difficult to achieve preoperatively from various imaging modalities.^3^, ^4^, ^5^ The gold standard for definitive diagnosis of gallbladder cancer is still pathological findings, and cholecystectomy is sometimes needed to attain total biopsy.^6^ In general, the diagnostic procedure should be as noninvasive as possible, and a laparoscopic approach for suspected gallbladder cancer appears reasonable in this respect.^7^ However, standard laparoscopic cholecystectomy remains risky in terms of exposing and spreading cancer cells during surgery.^8^

With the twin aims of both evaluating oncological safety and exploring lower invasiveness, we tested a novel approach for laparoscopic whole‐layer cholecystectomy (LWLC) and laparoscopic gallbladder bed dissection (LGBD) as total biopsy methods for suspected gallbladder cancer at our institution, and here, we report on the short‐term outcomes of a series of consecutive patients who underwent these procedures.

## 2 METHODS

This is a retrospective case series investigating clinicopathological data of laparoscopic total biopsy for suspected gallbladder cancer at Yamaguchi University Graduate School of Medicine. From September 2016 to July 2018, a laparoscopic approach was applied for consecutive patients with suspected gallbladder carcinoma based on preoperative ultrasonography (US), computed tomography (CT), magnetic resonance imaging (MRI), and fluorodeoxyglucose‐positron emission tomography (FDG‐PET). Preoperative imaging findings of suspected gallbladder cancer were defined as: elevated lesion >10 mm in diameter,^9^, ^10^ increasing tumor size over time compared with the previous imaging,^9^ sessile lesion,^9^, ^10^ irregular wall thickness lesion mimicking cancer,^3^ elevated lesion with dense enhancement,^11^, ^12^ or positive accumulation on FDG‐PET^13^, ^14^ (defined as an ^18^F‐FDG maximum standardized uptake value >3.65). To minimize the false negative rate in imaging diagnosis, patients with gallbladders that met at least one of the preceding findings identified by experienced radiologists at Yamaguchi University Graduate School of Medicine were eligible for the current study. Patients with gallbladder lesions located closely to the cystic duct, Glissonian sheath, and/or hepatoduodenal ligament were excluded.

**FIGURE 1:**
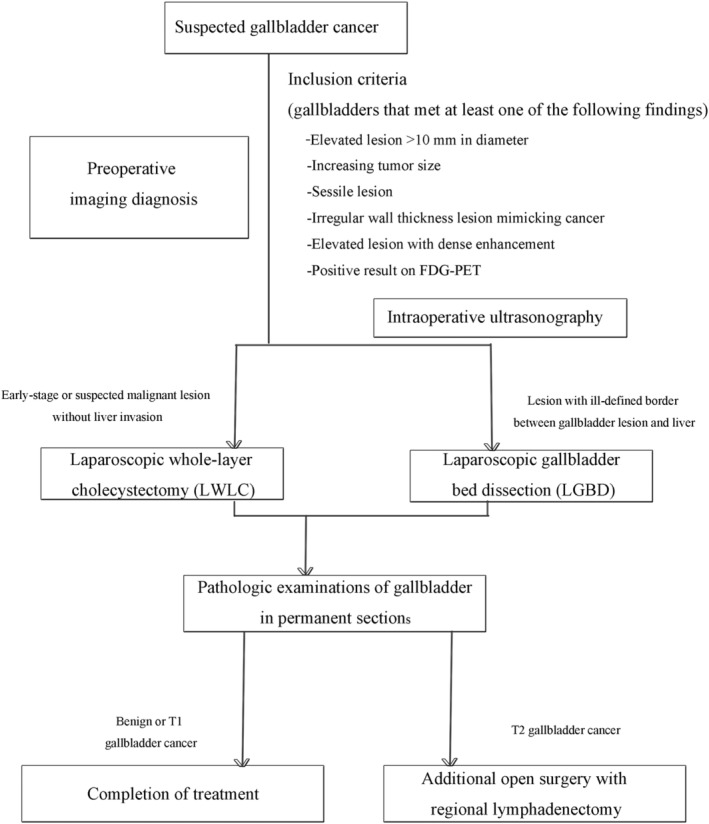
Algorithm for our laparoscopic approach to suspected gallbladder cancer. After laparoscopic total biopsy for suspected gallbladder cancer, pathologic examinations of permanent sections are performed for definitive diagnosis. When postoperative pathologic examination reveals pT2 gallbladder cancer, additional open gallbladder bed resection and regional lymphadenectomy are considered

The algorithm used for the laparoscopic approach to gallbladder lesions is shown in Figure 1. Intraoperative US was performed first during the operation. Contrast‐enhanced ultrasound, a reliable tool in the detection of focal liver lesions,^15^ was used to investigate whether the gallbladder lesion had a well‐ or ill‐defined border with the liver parenchyma. When an early‐stage or malignant lesion without liver invasion was suspected, LWLC was performed. When imaging showed an ill‐defined border between the gallbladder lesion and liver, LGBD was considered, and then the resection line of the liver was determined about 1 to 2 cm away from the gallbladder bed margin. We also intended to resect the lymph nodes around the cystic artery and cystic duct, including the sentinel lymph nodes. After laparoscopic resection, pathologic examination of the gallbladder in permanent sections was performed to achieve a definitive diagnosis.

When postoperative pathologic examination revealed the presence of gallbladder cancer invading into the subserosal layer (ie, pT2), D2 lymphadenectomy and additional gallbladder bed resection were considered as the second stage operation with curative intent. D2 lymphadenectomy is defined as removal of the lymph nodes in hepatoduodenal ligament with bile duct resection, around the common hepatic artery, and around the posterosuperior region of the pancreas head. This was performed as a routine operation in the additional surgery for pT2 cancer. After LWLC for a pT2 gallbladder cancer in contact with the liver, additional gallbladder bed dissection was performed to confirm negative margin. On the other hand, when negative margin of the gallbladder bed was proven by resected specimen during total biopsy surgery, such as a pT2 gallbladder cancer located only on the free peritoneal side and a pT2 gallbladder cancer resected with the gallbladder bed, only D2 lymphadenectomy was performed. The residual tumor status for the stump of cystic duct was also investigated carefully in permanent section, and additional bile duct resection was considered when pathologic findings of cystic duct were positive. These additional procedures were performed as open surgery.

Patient characteristics, perioperative findings, pathologic findings, and postoperative outcomes of patients who underwent LWLC or LGBD were reviewed retrospectively, and the short‐term outcomes of our laparoscopic approach were analyzed. All patients were followed‐up postoperatively until death or May 2019.

### 2.1 Statistical analysis

Background characteristics are presented as median and interquartile range (IQR) for continuous data, and as number and percentage for categorical data. Statistical analyses were performed using JMP version 13.0 software (SAS Institute Japan, Tokyo, Japan).

### 2.2 Ethical considerations

This study was approved by the institutional review board of Yamaguchi University Hospital (H2019‐009). Written informed consent was obtained from all patients.

## 3 RESULTS

Fifteen consecutive patients who underwent laparoscopic total biopsy for suspected gallbladder cancer between September 2016 and July 2018 were included in the study. The median age of the patients was 63 years (IQR 42‐76 years); 7 patients were males.

Short‐term outcomes for the 15 patients who underwent laparoscopic total biopsy for suspected gallbladder cancer are shown in Table 1. We performed LWLC in 12 cases and LGBD in 3 cases. Median operation time was 159 minutes (IQR 140‐193 min) and median blood loss was 10 mL (IQR 5‐30 mL). No bile leakage caused by intraoperative perforation of the gallbladder was encountered. Abdominal abscess without bile leakage, as a Clavien‐Dindo grade 3 complication, was observed in one patient after surgery (6.7%). Median postoperative hospital stay was 7 days (IQR 5‐9 days).

**TABLE 1:** Short‐term outcomes for patients who underwent laparoscopic total biopsy for suspected gallbladder cancer (n = 15)

Operation methods	
LWLC	12 cases
LGBD	3 cases
Median operation time (IQR)	159 min (140‐193)
Median blood loss (IQR)	10 mL (5‐30)
Complications	
Clavien‐Dindo classification (Grade III)	1 case (abscess)
Median postoperative hospital stay (IQR)	7 days (5–7)

Abbreviations: IQR, interquartile range; LGBD, laparoscopic gallbladder bed dissection; LWLC, laparoscopic whole‐layer cholecystectomy.

Clinicopathological features of the 15 patients are shown in Table 2. Based on our inclusion criteria, at least one preoperative imaging finding suggestive of malignancy was observed in each case, with an elevated lesion >10 mm in diameter found in eight cases, increasing tumor size in one case, a sessile lesion in one case, irregular wall thickness lesion mimicking cancer in six cases, an elevated lesion with dense enhancement in 11 cases, and FDG‐PET‐positive in four cases.

**TABLE 2:** Clinicopathological features of the 15 patients who underwent laparoscopic total biopsy for suspected gallbladder cancer

Case	Age	Sex	Preoperative imaging findings	Op	Findings of resected specimens						Other
					Macroscopic type	Size (mm)	No.	Location		Pathologic diagnosis	
1	68	F	Wall thickness, dense enhancement, PET‐positive	LGBD	Nodular type	20	1	Gfb	hep	Cancer (pT2)	Simultaneous pulmonary metastases, thyroid cancer
2	62	F	Wall thickness, dense enhancement	LGBD	Flat type	10	1	Gf	hep	Cancer (pT2)	
3	85	F	Wall thickness, dense enhancement	LWLC	Wall thickness	10	1	Gn	circ	Chronic cholecystitis with ADM	
4	73	M	>10 mm, dense enhancement	LGBD	Papillary type	22	1	Gf	hep	Cancer (pT1a‐RAS[SS])	
5	79	M	Wall thickness, dense enhancement	LWLC	Ulcer	12	1	Gbn	hep	Chronic cholecystitis with ulcer	
6	53	F	>10 mm, dense enhancement	LWLC	Is polyp	10	1	Gf	perit	Tubular adenoma	
7	74	F	>10 mm, sessile, dense enhancement, PET‐positive	LWLC	Papillary type	25	1	Gf	perit	Cancer (pT2)	
8	41	F	>10 mm, dense enhancement	LWLC	Is polyp	11	1	Gf	hep	Cholesterol polyp	
9	76	M	>10 mm, increasing tumor	LWLC	Isp polyp	5	M	Gb	hep	Cholesterolosis	
10	42	M	Dense enhancement	LWLC	Isp polyp	7	1	Gf	perit	Papilotubular adenoma	
11	80	M	>10 mm, dense enhancement, PET‐positive	LWLC	Papillary type	18	1	Gf	perit	Cancer (pT1a)	
12	40	M	>10 mm	LWLC	Isp polyp	12	M	Gbn	circ	Cholesterolosis	
13	40	F	>10 mm	LWLC	Isp polyp	13	2	Gf	hep	Cholesterol polyp with epithelial hyperplasia	
14	63	F	Wall thickness	LWLC	Wall thickness	12	1	Gf	perit	ADM	
15	60	M	Wall thickness, dense enhancement, PET‐positive	LWLC	Wall thickness	50	1	Gbf	circ	XGC	Postoperative abdominal abscess

Abbreviations: ADM, adenomyomatosis; circ, circumferential type; Gbn, body and neck of gallbladder, Gf, fundus of gallbladder, hep, hepatic side; Is, sessile; Isp, subpedunculated; LGBD, laparoscopic gallbladder bed dissection; LWLC, laparoscopic whole‐layer cholecystectomy; M, multiple; No., Number of lesion; Op, operation; perit, peritoneal side, XGC, xanthogranulomatous cholecystitis.

**FIGURE 2:**
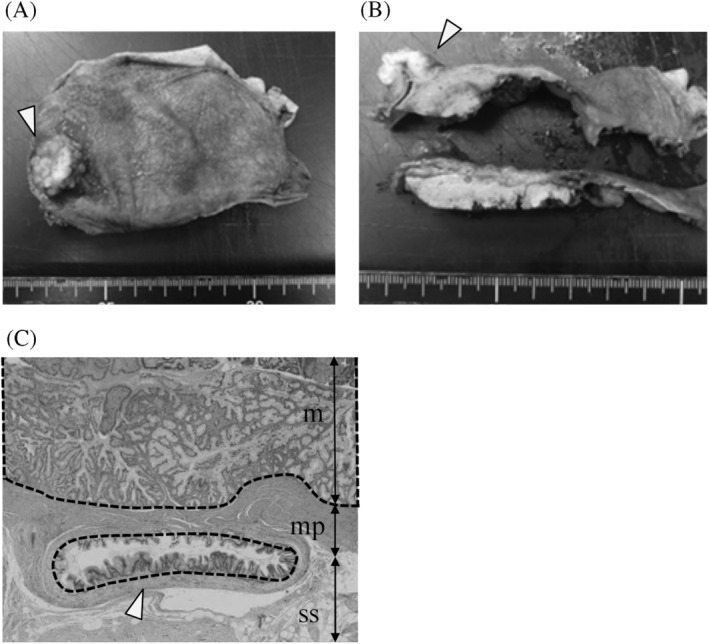
Postoperative pathologic findings in a patient diagnosed with pT1a‐RAS(ss) (Case 4) after LGBD. (A,B) Macroscopic findings: An elevated lesion (arrowhead) is observed in the fundus of the gallbladder. C, Microscopic findings: Mucosal cancer (dotted frame) in the Rokitansky‐Aschoff sinus (arrowhead) is observed in the subserosal layer. (HE stain, ×40)

Histologically, gallbladder tumors were diagnosed in seven cases (adenoma, n = 2; adenocarcinoma, n = 5). Among these seven patients, gallbladder cancer was diagnosed in 5 cases (pT1a, n = 2; pT2, n = 3), and two of the three pT2 patients underwent additional open surgery (Table 3). There were no patients with positive cystic duct margin of gallbladder cancer. In the patients with cancer, sentinel lymph nodes were also resected, in Case 4, 7, and 11 during total biopsy, and all of them were proven pathologically negative in permanent section. Although sentinel lymph nodes were not obtained in Case 1 and 2 during the total biopsy operation, all lymph nodes of Case 2 resected during additional operation were examined using permanent section and proven negative histopathologically. Case 1, which showed both thyroid cancer and gallbladder lesions suspicious of malignancy with simultaneous pulmonary metastases which were too small to obtain diagnostic tissue, received LGBD to evaluate whether the pulmonary metastases could have originated from the gallbladder, and pathologic examination demonstrated pT2 gallbladder cancer. In this case, additional surgery was not performed because of the distant metastases, and she died approximately 9 months after surgery due to progression of lung metastases. All other cases of gallbladder cancer remained alive without recurrence as of final follow‐up, with a median follow‐up of 13 months (IQR 8‐17). Postoperative pathologic findings in a patient diagnosed with pT1a‐RAS(ss) (Case 4) after LGBD, which involved complete removal of the cancer that was considered curative, are shown in Figure 2.

**TABLE 3:** Final pathologic findings of surgical specimens in the 5 patients diagnosed with gallbladder cancer

Case	Additional surgery	pT	pN	M	Histological type	ly	v	ne	Stage	Curability	Outcome (months of follow‐up)
1	(−)	T2	NX	PUL	Tub1	ly1	v1	ne3	IV	R2	Dead (9)
2	Lymphadenectomy with bile duct resection	T2	N0	(−)	Tub1	ly1	v1	ne0	II	R0	Alive (28), No recurrence
4	(−)	T1a	N0	(−)	Tub1	ly0	v0	ne0	I	R0	Alive (21), No recurrence
7	Lymphadenectomy with bile duct resection	T2	N0	(−)	Pap	ly0	v0	ne0	II	R0	Alive (18), No recurrence
11	(−)	T1a	N0	(−)	Pap	ly0	v0	ne0	I	R0	Alive (15), No recurrence

Abbreviations: ly, lymphatic invasion; M, distant metastasis; ne, perineural invasion; Pap, Papillary adenocarcinoma; pN, pathological assessment of the regional lymph nodes; pT, pathological assessment of the primary tumor stage; Tub1, Tubular adenocarcinoma, well‐differentiated; v, venous invasion;

## 4 DISCUSSION

This study shows that our laparoscopic approach to suspected gallbladder cancer may represent a safe and useful procedure for determining the optimal treatment strategy based on accurate T staging obtained histopathologically. Although various imaging modalities have been developed recently, including multidetector‐row CT,^16^, ^17^ MRI,^18^, ^19^ and endoscopic US,^4^, ^20^ exact preoperative diagnosis of gallbladder cancer remains difficult. Further, exact preoperative determination of the depth of tumor invasion (T stage), which is recognized as the most important prognostic factor and a crucial piece of information for selecting the optimal treatment strategy, is more difficult.^4^ Thus, patients presenting with radiologically suspicious gallbladder lesions might not always receive optimal surgery when imaging diagnosis alone is used for treatment planning. According to our algorithm, pathologic findings including depth of cancer invasion in the total biopsy specimen in permanent section, which provides information for making treatment decisions, can be obtained using minimally invasive procedures. Although intraoperative frozen tissue diagnosis is fairly reliable in determining whether lesions are malignant or benign,^21^, ^22^ the accuracy of frozen‐section diagnosis in terms of the depth of cancer invasion could be limited.^22^ In this respect, we decide the appropriate surgical strategy depending on the final pathologic diagnosis, including depth of cancer invasion, from permanent sections made after total biopsy.

As mentioned above, pT stage is the most important prognostic factor because the depth of gallbladder cancer invasion reflects lymphatic, perineural, and vascular invasion.^23^ Prognosis is good for patients diagnosed with pT1a carcinoma, and additional resection is not necessary if the surgical margins are negative.^8^ Although additional resection for patients with pT1b remains controversial, the fact remains that a small number of pT1b patients show positive lymph node metastasis.^8^, ^24^ Additional radical resection including regional lymphadenectomy is recommended in patients with pT2 or more advanced gallbladder cancer because positive lymph node metastasis could be observed at high rates.^8^, ^25^, ^26^, ^27^ For accurate pT stage diagnosis, complete resection of the gallbladder wall adjacent to the liver under safe procedures is of key importance in the first‐stage operation. In conventional laparoscopic cholecystectomy, the dissection layer of the gallbladder wall on the liver side is the subserosal layer. In this procedure, GBC in which the depth of invasion extends to the serosal layer, or even mucosal carcinoma in the Rokitansky‐Aschoff sinus, may result in positive surgical margins.^28^ LWLC and LGBD can avoid the risk of regional residual disease in the gallbladder wall adjacent to the liver, even in pT1a‐RAS(ss) cancer potentially cured by complete resection (Figure 2). Moreover, the need for additional operative treatments should be determined through meticulous microscopic investigation of the specimen, with special attention given to the depth of invasion. In the current study, additional radical operations were successfully performed for two of the three patients with pT2 gallbladder cancer, with no microscopic residual cancer or lymph node metastasis in the final pathologic findings. Both patients remained alive without recurrence as of last follow‐up. We demonstrated that our algorithm for suspected gallbladder cancer could facilitate determination of the appropriate treatment option according to the exact pT stage.

The pathologic status of the stump of cystic duct is also very important. Intraoperative pathologic diagnosis using frozen section of the stump of cystic duct is often performed during cholecystectomy for suspected gallbladder cancer; however, preliminary results based on frozen section analysis can be difficult to interpret, and the accuracy of frozen‐section diagnosis may be unreliable in the setting of acute inflammation.^29^ In this respect, we had not used intraoperative pathologic diagnosis of the stump of cystic duct since gallbladder lesions closely located at the cystic duct were excluded in the current study. There were no patients with positive cystic duct margin of gallbladder cancer in the current study. Moreover, recently, Ethun et al^29^ reported that too short (before 4 wk) and too long (after 8 wk) time intervals from the initial cholecystectomy to reoperation, R2 resection, and advanced T stage of the patients who underwent re‐resection for incidentally discovered gallbladder cancer were associated with worse survival on multivariable analysis; however, the presence of residual disease at reoperation was not associated with worse survival on multivariable analysis. Thus, we consider that this total biopsy approach should be used in patients whose gallbladder lesion is sufficiently distant from the cystic duct (as judged by preoperative imaging)— to avoid the case in which the operation is performed while invasive cancer still remains in the cystic duct—, and residual cancer in stump of cystic duct should be carefully examined in a permanent section of postoperative pathologic diagnosis. Additional bile duct resection should be planned within the optimal time interval for re‐resection in patients with gallbladder cancer when pathologic findings of cystic duct mucosa are positive.

During laparoscopic surgery, special attention should be paid to preventing bile spillage, as this event might induce cancer cell dissemination and trocar site metastasis^30^, ^31^ Gallbladder perforation during LC is strongly associated with recurrence and worse patient survival,^1^, ^26^ and whether a laparoscopic approach is acceptable for suspected gallbladder cancer remains controversial. Previously, laparoscopic surgery was considered contraindicated for suspected gallbladder cancer because of the increased incidence of peritoneal dissemination and port‐site recurrence.^30^, ^31^ In contrast, recent reports have found no association between LC and worse prognosis when comparing survival rates with patients undergoing a standard open surgical procedure, as long as additional excision was conducted for patients with pT2 or pT3 gallbladder cancer.^6^, ^32^ LC is now performed by many surgeons and is considered a reliable technique for gallbladder lesions even in cases of possible malignant lesions^33^; however, safe and robust techniques need to be developed for widespread adoption of laparoscopic total biopsy of suspected gallbladder cancer. In the current study, no bile spillage resulted from intraoperative perforation of the gallbladder, and we believe that LWLC and LGBD can reduce the risks of intraoperative perforation and excessive manipulation of the gallbladder. In addition, a laparoscopic approach avoids unnecessary open surgery in those patients finally confirmed as having benign lesions. Some patients with preoperatively suspected gallbladder cancer can be diagnosed with benign gallbladder diseases such as adenomyomatosis or xanthogranulomatous cholecystitis in the final postoperative pathologic findings.^4^, ^24^ Moreover, simple cholecystectomy alone for patients with pT1a GBC can achieve satisfactory surgical results.^24^, ^25^ In the present 15 cases, eight had nonneoplastic lesions in the gallbladder, two had adenoma, and one had T1a cancer that could be cured with simple cholecystectomy, with only one case of pT1a‐RAS(ss). All of these cases were diagnosed using a laparoscopic approach, and unnecessary open surgery was able to be avoided.

In the present study, our laparoscopic approach achieved good short‐term outcomes, although limitations include the fact that this is a case series, with a small number of patients and a short follow‐up period. We believe that this procedure may offer a feasible method for achieving total biopsy of suspected gallbladder cancer and may have an important role to play in helping determine treatment strategies depending on the stage of gallbladder cancer. Since August 2018, the Yamaguchi Pancreatic/Biliary Disease Study Group has been conducting a prospective observational study to assess the safety and feasibility of these methods in a larger sample, in the Laparoscopic Approaches for suspected GallBladder cancer in Yamaguchi study (LAGBY study: UMIN000035352).

## CONFLICT OF INTEREST

The authors declare no conflict of interest for this article.

## AUTHOR CONTRIBUTIONS

Conceptualization: Yukio Tokumitsu, Hiroaki Nagano

Data Curation: Satoshi Matsukuma, Masao Nakajima

Methodology: Shin Yoshida, Michihisa Iida, Nobuaki Suzuki, Shigeru Takeda

Writing — Original Draft Preparation: Yukio Tokumitsu

Writing — Review & Editing: Yukio Tokumitsu, Yoshitaro Shindo, Hiroto Matsui, Hiroaki Nagano

All authors have read and approved the final version of the manuscript. Yukio Tokumitsu had full access to all of the data in this study and takes complete responsibility for the integrity of the data and the accuracy of the data analysis.

## TRANSPARENCY STATEMENT

The lead author (Yukio Tokumitsu) affirms that this manuscript is an honest, accurate, and transparent account of the study being reported; that no important aspects of the study have been omitted; and that any discrepancies form the study as planned (and, if relevant, registered) have been explained.

## Data Availability

The datasets used during the present study are available from the corresponding author on reasonable request.
